# Crisis in the Family and Positive Youth Development: The Role of Family Functioning

**DOI:** 10.3390/ijerph16101678

**Published:** 2019-05-14

**Authors:** Jaroslava Mackova, Zuzana Dankulincova Veselska, Daniela Filakovska Bobakova, Andrea Madarasova Geckova, Jitse P. van Dijk, Sijmen A. Reijneveld

**Affiliations:** 1 Department of Health Psychology, Medical Faculty, PJ Safarik University, Trieda SNP 1, 040 11 Kosice, Slovak Republic; zuzana.dankulincova@upjs.sk (Z.D.V.); daniela.filakovska@upjs.sk (D.F.B.); andrea.geckova@upjs.sk (A.M.G.); 2 Graduate School Kosice Institute for Society and Health, PJ Safarik University, Trieda SNP 1, 040 11 Kosice, Slovak Republic; j.p.van.dijk@umcg.nl; 3 Olomouc University Social Health Institute, Palacky University in Olomouc, Univerzitni 22, 771 11 Olomouc, Czech Republic; 4 Department of Community & Occupational Health, University Medical Center Groningen, University of Groningen, Antonius Deusinglaan 1, 9713 AV Groningen, The Netherlands; S.A.Reijneveld@umcg.nl

**Keywords:** positive youth development, crisis in the family, family activities, parental supervision, adolescents

## Abstract

The family is a very important institution that provides relationships and contexts in which adolescents are included and where the trajectory of positive development can be activated. A family crisis can affect family functioning and endanger adolescent development. Therefore, we aimed to explore the association of crisis in the family with positive youth development (PYD), and further, whether adolescent-perceived family functioning mediates or moderates this relation. The sample consisted of Slovak adolescents (*N* = 341, 44% boys, mean age = 13.16) who completed questionnaires that included questions on family crisis and joint family activities, the Alabama parenting questionnaire and the Very Short PYD questionnaire in the baseline measurement of the Care4Youth cohort study. We found a positive association of perceived positive parenting (B = 0.51; *p* < 0.001) and family activities (B = 0.50; *p* < 0.001) with PYD, whereas crisis in the family (B = −0.42; *p* = 0.01) and perceived poor supervision (B = −0.30; *p* < 0.001) were negatively associated with PYD. Using serial mediation model, we found following pathway which connected crisis in the family with PYD: crisis in the family → perceived poor parental supervision → joint family activities → PYD. This implies that family interventions and counselling to support parenting skills, especially parental supervision and family activities, to those with the signs of an ongoing family crisis may help to counteract the negative effect of the family crisis on PYD.

## 1. Introduction

The positive youth development (PYD) theory regards development as a process of growth and increasing competences [1] and of maintaining the capacity and potential [2] in every child, including those from the most disadvantaged backgrounds [3]. This growth, according to the PYD theory, is focused on optimal development of youth in five domains: competence, confidence, connection, character and caring. For competence, this is a positive view of one’s actions in specific areas—in the social area it refers to interpersonal skills, in the cognitive area to cognitive abilities, in the academic area to school performance and academic achievements, and in the working area to work habits and the ability to explore career choices. For confidence, positive development is an internal sense of positive self-worth and self-efficacy. For connection, this refers to positive bonds between adolescents and other people and important institutions, including family, peers, school and community. For character, positive development is connected to moral development of youth and refers to the integrity of individuals, respect for norms, a sense of right and wrong. For caring, it means empathy for other people [4,5]. An important idea which is included in PYD theory is that “being problem free is not fully prepared” [6], which means that the reduction or removal of risk behaviour in adolescents is not enough for their healthy development. The main goal for PYD is a fully developed adolescent—socially, morally, emotionally, physically and cognitively [7].

PYD theory is rooted in the relational developmental system theory, which is centred on the idea that the essential strength of human development is plasticity. This plasticity is provided by a complex system of relationships between individuals and their contexts [8]. One of the main principles of PYD is that the trajectory of PYD is activated when adolescents are involved in relationships, contexts and environments that facilitate their development [2]. The family is a very important environment that provides relationships and contexts in which adolescents are included, and where the PYD trajectory can be activated. The Search Institute in Minneapolis [9] has identified 40 developmental assets that are helpful in the positive development of adolescents. These assets include important factors connected to family, such as family support, family communication, parent involvement in schooling, family boundaries, adult role models, high expectations and time spent at home. Notably, the 4-H study (e.g., [5]), revealed that having dinner together as a family was one of the most important factors associated with PYD [10]. Similarly, the time that family spent together was also associated with positive outcomes in youth, such as educational attainment and post-school labour market position [11]. Finally, parental knowledge of the daily activities of their offspring has been shown to be an important predictor of various positive outcomes [12].

Family crises, such as conflicts between parents, divorce or substance abuse, may endanger the healthy development of adolescents. Previous studies have explored associations of specific forms of family crisis with a wide range of developmental outcomes. Children exposed to violence between their parents were found to have higher odds of risk-taking behaviour in adolescence [13] and were at higher risk of developing emotional and behavioural problems [14]. Divorce or living in an incomplete family was connected with lower subjective well-being [15], a higher level of anxiety and depression [16], a higher likelihood of emotional and behavioural problems [17] and a higher risk of drunkenness and frequent alcohol drinking [18]. Alcohol use and abuse of at least one parent was related to a greater risk for aggressive and delinquent behaviour [19] and a greater increase in alcohol use in adolescence [20]. Moreover, family crises were found to be associated with lower self-esteem in adolescents [21,22,23], worse development of social competence [24], worse academic achievement [25,26,27] and worse school adjustment [28]. However, evidence on the association between any experienced crisis in the family and PYD as an overall concept is still scarce.

We hypothesize that these crises in families can disrupt developmental assets, causing the trajectory of PYD to not be activated. Research also suggests that various family crises are connected to worse family functioning, including poor parenting, disciplining and parental supervision and fewer joint family activities. For example, family conflicts have been found to be related to worse maternal and paternal monitoring of adolescents [29]. Chassin et al. [30] found that alcohol-abusing parents knew less about their offspring’s daily activities. Moreover, King and Chassin [31] found that children of alcohol-abusing parents received less disciplining from their parents. Research also suggests that crisis in a family can negatively affect joint family activities that are one of the most important indicators of family functioning [32,33].

Research suggests that aspects of family functioning could have a mediating or moderating role in the association between family crisis and adolescents’ developmental outcomes. Worse family functioning is not only associated with family crises but has also been shown to be connected with various negative developmental outcomes in adolescents. For instance, inconsistent discipline was associated with adjustment problems [34], and adverse parenting during childhood was related to psychopathology in adulthood [35]. Moreover, family functioning has been shown to mediate the association between interparental conflict and adolescent depressed mood [36] and the association between parental drinking and adolescent substance use and problem behaviour [37]. This mediating role of family functioning can be explained by the Family stress model [38,39]. The Family stress model explains how family stressors (especially economic hardship) lead to worse parenting via parental psychological distress, and that worse parenting, in turn, leads to worse developmental outcomes in children. It seems likely that this pathway also applies to other crises in the family and to development outcomes in children or adolescents via parenting practices and family functioning, in the same way as for economic hardship.

On top of having a mediating role, family functioning might also be a protective factor; for example, a moderating role of family functioning in the association of parental problem drinking and children’s adjustment was found [40]. Additionally, family meals may be a protective factor for substance use in adolescents [41], and positive parenting may be a protective factor of antisocial behaviour in deprived children [42]. Next, consistent disciplining has been shown to moderate the relation between divorce stressors and internalizing and externalizing problems in children [43]. However, previous research was focused on specific types of family crisis and did not asses the overall effect of crises. Moreover, it did not use PYD as a developmental outcome.

Therefore, the aim of this study was first to explore the association of experienced crisis in the family with PYD, and second whether family functioning mediates or moderates this relation.

## 2. Materials and Methods

### 2.1. Sample

We used data from the baseline measurement wave of the Care4Youth cohort study. Participants were recruited via randomly chosen primary schools in the Kosice region in eastern Slovakia, which were approached from January until June 2017 using a two-stage sampling process. In the first stage, we contacted 11 primary schools, seven of which took part in our survey (response rate 64%). All schools were located in Kosice, the second largest city in Slovakia, and were mostly attended by children from families with a middle or high socioeconomic status. Six schools were state primary schools, four of them with attendances ranging between 557 and 687 children and two with attendances of 276 and 171 children. One school was private, with the attendance of 147 children. In the second stage, 1599 parents or legal representatives of pupils were contacted. After being thoroughly informed about the potential risks and consequences of participation in the study, parents were asked to provide us with a signed informed consent on behalf of their children and themselves (response rate 23.4%). We obtained data from a sample of 341 adolescents from the 5th to 9th grades and aged from 10 to 16 years old (response rate 94.3%, mean age 13.14 years, 44% boys).

### 2.2. Procedure and Measures

Adolescents filled in the questionnaires in the absence of teachers during regular class time, with assistance from researchers and trained research assistants, if needed.

Positive youth development (PYD) was measured by the Very Short Measure of PYD, which was adapted in collaboration with the authors of the original measure [44], i.e., one item of the original version was replaced by a more culturally appropriate item (item “Some teenagers feel that they are better than others their age in sports” was replaced with the item from the Rosenberg self-esteem scale “I am able to do things as well as most other people”) based on pilot testing, and the answering format of all questions was unified. The final version consisted of 17 items assessed on a 5-point Likert scale from strongly disagree to strongly agree. This questionnaire measures the five Cs of PYD: competence (item example: “I do very well in my class”), confidence (item example: “All in all, I am glad I am me”), connection (item example: “In my family, I feel useful and important”), caring (item example: “When I see someone being taken advantage of, I want to help them”) and character (item example: “I think it is important to accept responsibility for my actions when I make a mistake or get in trouble”). Scores on these five Cs load on a higher-order PYD latent construct as a single indicator of PYD [45]. In line with these findings, we use the standardized aggregate score of the five Cs as a dependent variable; a higher score indicates a more positive development. Cronbach’s α in our sample was 0.85.

Crisis in the family was measured using the following questions: “Have you ever experienced some of the following events? 1. Death of your father or mother? 2. Problems with alcohol or drugs of one of your parents? 3. Serious conflicts or physical fights between your parents? 4. The divorce of your parents?”. Questions could be answered by Yes/No. A positive answer for at least one question was classified as there being a crisis in the family (1), whereas instances in which all answers were negative were classified as there being no crisis in the family (0).

Family functioning was measured via adolescent-perceived parenting style (parenting, discipline, supervision) and family activities. Perceived parenting style was measured by the Alabama Parenting Questionnaire—short version [46], which consists of nine items divided into three subscales: positive parenting (item example: “How often do your parents tell you that you are doing a good job with something?”), poor supervision (item example: “How often do you fail to leave a note or let your parents know where you are going?”) and inconsistent discipline (item example: “How often do you talk your parents out of punishing you after you have done something wrong?”). Each subscale consisted of three items that were assessed on a 5-point Likert scale from “never” to “always”. A higher score in all subscales indicates the measured aspect of parenting style to be more prevalent. Cronbach’s α for each subscale ranged from 0.58 to 0.80, with an acceptable MIIC (mean inter-item correlation) ranging from 0.32 to 0.59. In the analyses we used standardized scores per subscale.

Family activities [11] were assessed by the following questions: 1. “How many days in a week do you usually have dinner with your parents (or one of them, or with the adults you live with)?” and 2. “How many days in a week do you usually talk with your parents (or one of them or adults you live with) about your stuff?” Possible answers for both questions were (0) “Never”, (0) “Rarely”, (1) “Most days”, (1) “Every day”. The last question was: 3. “How often do you and your parents (or one of them, or adults you live with) do something together? For example, you go to the cinema, for a walk, take a trip, visit family or attend some sports events and so on?” The possible answers were (0) “Almost never”, (0) “About once a year”, (0) “Several times a year”, (0) “About once a month”, (1) “About once a week”, (1) “More than once a week”. The sum score of these three items was then calculated; the higher the score, the more prevalent the family activities. In the analyses we used the standardized score.

Perceived socioeconomic status of the family (SEP) [47] was measured as a possible cofounder on a 10-point “ladder” scale (0—the worst, 10—the best). Children were asked to assess where they see their families on this ladder according to their socioeconomic position—how much money they have, what level of education their parents achieved and how profitable the work of their parents is. A higher score indicates a higher perceived socioeconomic status.

### 2.3. Statistical Analysis

First, we described the background characteristics of the sample using descriptive statistics. Second, we performed a series of analyses to explore the associations of crisis in the family and of family functioning with PYD, using linear regression analysis. We repeated these analyses with adjustment for gender, age and perceived socioeconomic status of the family. In these exploratory analyses, we also examined the moderating effect of perceived positive parenting, perceived poor supervision and family activities on the association between crisis in the family, and PYD was examined by adding in each of the listed interactions (perceived positive parenting × crisis, perceived poor supervision × crisis, and family activities × crisis) separately into a regression model. Next, based on this exploration we conducted final analyses on the mediation by family functioning of the relation between crisis in the family and PYD for all respondents. We did so by assessing the mediation effect of all variables separately and then building a serial mediation model using the PROCESS macro model 6 [48]. These analyses were all controlled for gender, age and SEP, and all indirect effects were subjected to follow-up bootstrap analyses, with 5000 bootstrap samples and 95% bias-corrected confidence intervals. All statistical analyses were performed in SPSS v 23 (IBM Corpotation, New York, NY, USA) for Windows.

## 3. Results

Table 1 shows the descriptive statistics on crisis in the family, perceived family functioning and PYD for the study sample.

In the exploratory analyses, as reported in the Supplementary files (Tables S1 and S2), we found that the association of family crisis and PYD was significant; an occurrence of at least one type of crisis in a family was negatively associated with PYD (B = −0.42 **). All assumed mediators, except inconsistent disciplining, were also significantly associated with PYD; perceived positive parenting (B = 0.51 ***) and more frequent parental activities (B = 0.50 ***) were positively associated with PYD, while perceived poorer parental supervision (B = −0.30 ***) was negatively associated with PYD (Table S1). Crisis in the family remained significantly associated with PYD (B = −0.29 *) even after adjustment for gender, age and perceived socioeconomic status of the family (Table S2), but this association lost its significance (B = −0.13) after adding variables indicating family functioning (idem, Multivariate model 2). In the exploratory linear regression analyses, we next assessed the moderation effect of perceived positive parenting, perceived poor supervision and family activities on the relation between crisis in the family and PYD. This showed that none of these possible moderations was statistically significant.

**Figure 1:**
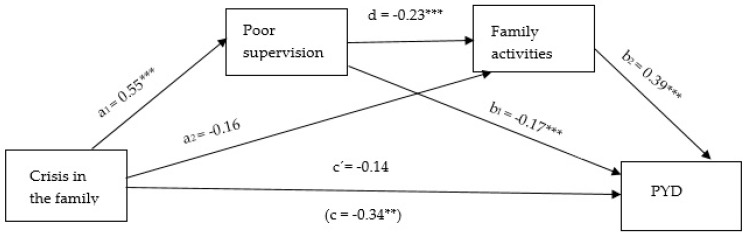
Serial mediation by poor supervision and few family activities of the relation between crisis in the family and PYD. Notes: * *p* < 0.05, ** *p* < 0.01, *** *p* < 0.001. All presented effects are unstandardized; a_n_ is the effect of crisis in the family on mediators; b_n_ is the effect of mediators on PYD; c’ is the direct effect of crisis in the family on PYD, and c is the total effect of crisis in the family on PYD; d is the effect of poor supervision on family activities.

Next, we assessed mediation effect of each presumed mediator in the association of crisis in the family and PYD separately; Figure S1 shows mediation effect of positive parenting, which was nonsignificant; Figure S2 shows mediation effect of poor parental supervision and Figure S3 shows mediation effect of family activities, that both were significant. The final serial mediation analyses show that crisis in the family is indirectly associated with PYD through its relation with poor supervision and family activities. As Figure 1 shows, adolescents who experienced crisis in their families reported worse parental supervision (a_1_ = 0.55 ***), and poorer supervision was associated with fewer family activities (d = −0.23 ***) which was negatively related to PYD (b_2_ = 0.39 ***). A 95% bias-corrected confidence interval based on 5000 bootstrap samples indicated that the indirect effect through poor supervision and family activities was entirely below zero; the estimate was −0.049 and the interval (−0.097 to −0.017).

## 4. Discussion

The aim of this study was to examine the role of perceived family functioning in the association between crisis in the family and PYD. We found that crisis in the family and adolescent-perceived poor parental supervision were negatively associated with PYD, whereas perceived positive parenting and more frequent family activities were positively connected with the healthy development of adolescents. Moreover, the relation between crisis in the family and PYD was mediated by perceived poor supervision and fewer family activities.

We found that adolescents who experienced at least one crisis in the family scored lower in PYD. This finding is in line with previously published research, which confirmed associations of specific forms of a family crisis with a wide range of developmental outcomes [13,14,15,16,17,18,19,20,21,22,23,24,25,26,27,28,49,50,51]. The interpretation of this finding is in line with the PYD theory. Troubles in the family, such as alcohol abuse, violence or divorce, are in contrast with family assets [52], which are very important for PYD (especially nurturing relationships that include, for example, positive communication in the family or showing affection to each other).

We found that crises in the family are connected to worse supervision. In interpreting this finding we have to take into account the fact that our respondents were adolescents (not parents) and that questions focused on how often adolescents failed to let parents know where they were going, how often they stayed out after the time that they were supposed to be at home and how often they met friends that parents did not know. We can interpret responses to these questions in at least two ways. First, we can interpret them such that adolescents who experienced any form of crisis in the family perceived lower levels of parental supervision, which is in agreement with previous research [29,30]. Second, we can interpret this finding such that adolescents who experienced a crisis in the family are less willing to share information with their parents. Both perceived parental supervision and the willingness to disclose to parents have been shown to be connected with positive outcomes; for example, Steinberg et al., [53] found that adolescents who perceive higher levels of parental monitoring have friends who are less involved in deviant behaviour. Kerr et al., [54] found that voluntary disclosure was connected to healthy parent-adolescent relationships. Additionally, Keijsers et al., [55] found that adolescent disclosure was a negative predictor of delinquency. This suggests that both pathways may apply in this case.

We also found that the indirect effect of the association of family crisis with PYD via parental supervision is more complex. This pathway involves several steps: crisis in the family → perceived poor parental supervision → joint family activities → PYD. This may be interpreted as meaning that adolescents who experienced any family crisis reported less parental supervision, leading to them having more unsupervised time, staying outside home later than parents had allowed, and meeting friends that parents did not know about. These adolescents may be less willing to spend time with parents and to share information about their lives, or parents may be less willing to spend time with these adolescents. All of these factors may contribute to worse development of adolescents. No previous study has assessed this pathway, but our findings align with previously reported evidence. First adolescents who are less involved in disapproved leisure time activities have been shown to be more likely to disclose to their parents [56], which is an important factor contributing to good parent–child relationships [54], and in turn contributes to positive development. Moreover, research has shown that family activities are beneficial for adolescents’ emotional well-being, especially when fathers were present [57]. In the case of family crises, this may not always be possible. Family supervision and activities are thus important factors in the pathway form family crises to youth development.

Next, we found that the association between crisis in the family and PYD disappears when variables are added to the regression model that relate to family functioning; however, we found the same effect in mediation models. We can interpret this finding as meaning that the association between family crisis and PYD is fully mediated by family functioning, namely perceived poor supervision and few joint family activities. This means that family crisis is not necessarily a risk factor for youth development per se, but that poor parental supervision and few family activities, both of which are often connected to family crises, can endanger the development of adolescents.

In contrast to our expectations, we found that inconsistent discipline was not connected to PYD. Previous research has shown that inconsistent discipline plays an important role in developing delinquent behaviour and decreasing socially competent behaviour [58]. An explanation may be that these outcomes are different from PYD as assessed in our study. PYD includes not only abilities, such as problem solving and school adjustment, but also other domains related to caring about others, connection with others, self-efficacy and self-esteem, and these are probably related more to other aspects of parenting than discipline (such as parenting, joint family activities and supervision). Such aspects may be affected less by disciplining, and more by other more positive parenting behaviours.

Moreover, we found that perceived positive parenting does not mediate the association between family crises and PYD. While perceived positive parenting was associated with PYD, it was not associated with family crisis. An explanation may be that not every family crisis has to affect parenting, and that when adolescents have a problematic relationship with only one of the parents, they could report on the parenting of the less or non-problematic parent, and similarly in cases of the death of a parent or divorce. Strohschein reported similar findings [59] (i.e., that the parenting of divorced parents was not worse than the parenting of non-divorced parents). Also, Zwaluw et al. [60] found that parental problem drinking was not associated with parenting.

This study contributes to the currently discussed PYD theory with a more complex examination of the nature of the relation between crisis in the family, perceived poor supervision, family activities and PYD. This study also revealed a mediating effect of perceived poor supervision and family activities on the association between crisis in the family and PYD that has not been studied before. Moreover, we revealed that the pathway that connects family crisis with PYD may be more complex than assumed in the past (i.e., it seems as though crisis in the family leads to worse PYD via poor parental supervision, which leads to fewer joint family activities).

One of the strengths of this study is that it used a version of the PYD questionnaire adapted for the population of Slovak adolescents and which has good internal reliability. In addition, our analyses controlled for important variables (age, gender and perceived socioeconomic status) that could potentially influence the results. However, the following limitations should be mentioned. First, the cross-sectional design of the study does not allow us to confirm the causal relationship between the explored variables and PYD. Next, active parental consent was required in our study, and as a result, our response rate was quite low and could be a source of bias, with potentially more problematic adolescents being included to a lesser degree. However, a study by Dent et al., [61] indicated that even lower response rates due to the need for active parental consent do not have to result in underrepresentation of children with poorer mental health.

We found that poor supervision and joint family activities are important factors contributing to PYD. Moreover, we found that poor supervision is indirectly connected to PYD through family activities. This implies that family interventions and counselling to support parenting skills, especially parental supervision and family activities, to those with the signs of an ongoing family crisis may help to counteract the negative effect of the family crisis on PYD. With regard to future research, we need to investigate these mechanisms in a larger and more representative sample, including adolescents from problematic families in particular. Next, a larger sample may allow the effects of various types of family crises, such as divorce, alcohol or drug abuse, violence and conflicts between parents and death of one of parents, to be investigated separately. These crises all occur in families with adolescents, but their impacts may vary. Moreover, to further explore causality in these mechanisms, longitudinal study designs should be used.

## 5. Conclusions

This study examined the associations between crisis in the family and family functioning and PYD. The presence of a crisis in the family and perceived poor supervision are both negatively connected with the healthy development of adolescents, while perceived positive parenting and more frequent family activities are positively associated with the healthy development of adolescents. Moreover, poor supervision and joint family activities are the mechanisms which connect family crises with PYD. Interventions should thus focus on supporting these aspects of family functioning in particular.

## Figures and Tables

**Table 1:** Background characteristics of the sample (341 Slovak adolescents aged 10–16, collected in 2017).

Sample Characteristics		*N* (%)
Gender ^1^		
Female		191 (56.20)
Male		149 (43.80)
Crisis in the family ^2^		
Yes		83 (25.00)
No		249 (75.00)
	Range	Mean (SD)
Age ^3^	10–16	13.16 (1.45)
Perceived socioeconomic status of the family ^4^	1–10	7.15 (1.54)
PYD ^5^	21–83	65.09 (8.97)
Family functioning		
Perceived positive parenting ^6^	4–15	11.67 (2.32)
Perceived poor supervision ^7^	3–14	5.72 (2.52)
Perceived inconsistent discipline ^8^	3–15	7.83 (2.56)
Family activities ^9^	0–3	1.89 (1.02)

SD: standard deviation; PYD: positive youth development; ^1^ *N* = 340, ^2^ *N* = 332, ^3^ *N* = 336, ^4^ *N* = 337, ^5^ *N* = 316, ^6^ *N* = 336, ^7^ *N* = 329, ^8^ *N* = 319, ^9^ *N* = 339.

## Supplementary Materials

The following are available online at https://www.mdpi.com/1660-4601/16/10/1678/s1: Figure S1: The mediation effect of perceived positive parenting in the association of crisis in the family and PYD, Figure S2: The mediation effect of perceived poor supervision in the association of crisis in the family and PYD, Figure S3: The mediation effect of family activities in the association of crisis in the family and PYD, Table S1: Associations of crisis in the family and family functioning with positive youth development using linear regression leading to unstandardized regression coefficients (B) and 95% confidence intervals (CI) (316 Slovak adolescents aged 10–16, collected in 2017), Table S2: Associations of crisis in the family and family functioning with positive youth development using linear regression models, i.e., adjusted for gender, age and perceived socioeconomic position of the family (Model 1) and additionally for all variables (Model 2), leading to regression coefficients (B) and 95% confidence intervals (CI) (316 Slovak adolescents aged 10–16, collected in 2017).
