# Supplementary material for: Crisis in the Family and Positive Youth Development: The Role of Family Functioning
Source: Int J Environ Res Public Health. 2019 May 14;16(10):1678. doi: 10.3390/ijerph16101678 (PMC6571796; doi:10.3390/ijerph16101678)
Supplement: Supplementary file 1 [file ijerph-16-01678-s001.pdf]

Supplementary files

Table S1. Associations of crisis in the family and family functioning with positive youth development using linear regression leading to unstandardized regression coefficients (B) and 95% confidence intervals (CI) (316 Slovak adolescents aged 10–16, collected in 2017).

	**Univariate B (95% CI)**
Crisis in the family	−0.42 (−0.67; −0.16) **
Perceived positive parenting	0.51 (0.42; 0.61) ***
Perceived poor supervision	−0.30 (−0.40; −0.19) ***
Perceived inconsistent discipline	−0.09 (−0.20; 0.02)
Family activities	0.50 (0.40; 0.59) ***

*** $p < 0.001$, ** $p < 0.01$, * $p < 0.05$.

Table S2. Associations of crisis in the family and family functioning with positive youth development using linear regression models, i.e., adjusted for gender, age and perceived socioeconomic position of the family (Model 1) and additionally for all variables (Model 2), leading to regression coefficients (B) and 95% confidence intervals (CI) (316 Slovak adolescents aged 10–16, collected in 2017).

	**Multivariate Model 1 B (95% CI)** [a]	**Multivariate Model 2 B (95% CI)** [b]
Crisis in the family	−0.29 (−0.55; −0.04) *	−0.13 (−0.36; 0.10)
Perceived positive parenting		0.28 (0.17; 0.39) ***
Perceived poor supervision		−0.13 (−0.23; −0.02) *
Perceived inconsistent discipline		−0.02 (−0.12; 0.08)
Family activities		0.29 (0.18; 0.40) ***
R-square	0.12 ***	0.40 ***

*** $p < 0.001$, ** $p < 0.01$, * $p < 0.05$; [a] Adjusted for gender, age and perceived socioeconomic status; [b] Adjusted for gender, age and perceived socioeconomic status and all other variables in the model.

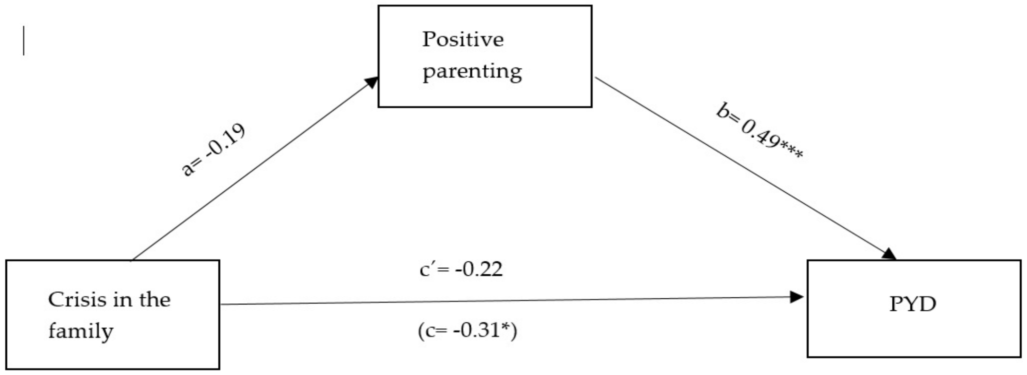

Figure S1. The mediation effect of perceived positive parenting in the association of crisis in the family and PYD. Notes: * $p < 0.05$, ** $p < 0.01$, *** $p < 0.001$. All presented effects are unstandardized; a is the effect of crisis in the family on positive parenting; b is the effect of positive parenting on PYD; c′ is the direct effect of crisis in the family on PYD, and c is the total effect of crisis in the family on PYD.

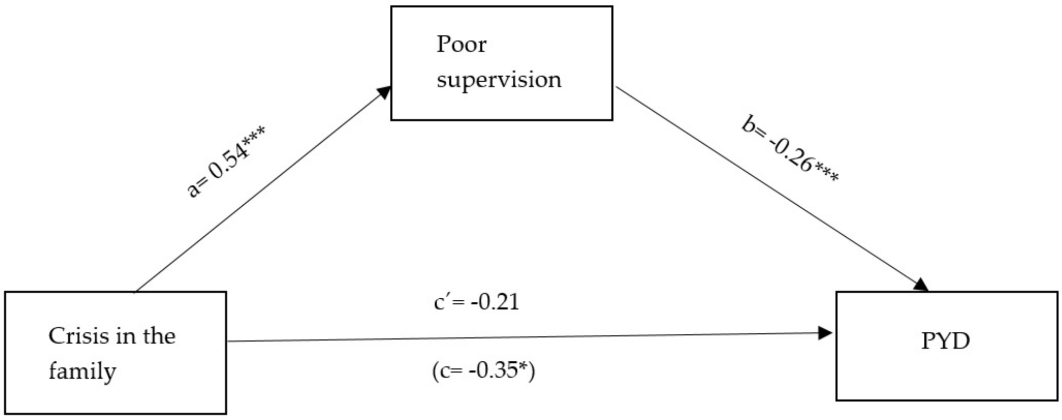

Figure S2. The mediation effect of perceived poor supervision in the association of crisis in the family and PYD. Notes: * $p < 0.05$, ** $p < 0.01$, *** $p < 0.001$. All presented effects are unstandardized; a is the effect of crisis in the family on poor supervision; b is the effect of poor supervision on PYD; c′ is the direct effect of crisis in the family on PYD, and c is the total effect of crisis in the family on PYD.

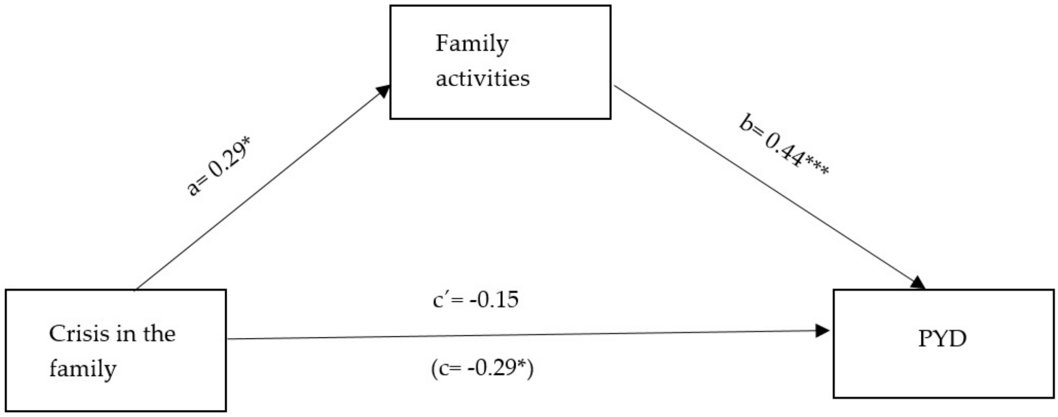

Figure S3. The mediation effect of family activities in the association of crisis in the family and PYD. Notes: * $p < 0.05$, ** $p < 0.01$, *** $p < 0.001$. All presented effects are unstandardized; a is the effect of crisis in the family on family activities; b is the effect of family activities on PYD; c′ is the direct effect of crisis in the family on PYD, and c is the total effect of crisis in the family on PYD.
